# Detecting Outlier Microarray Arrays by Correlation and Percentage of Outliers Spots

**Published:** 2007-02-24

**Authors:** Song Yang, Xiang Guo, Yaw-Ching Yang, Denise Papcunik, Caroline Heckman, Jeffrey Hooke, Craig D. Shriver, Michael N. Liebman, Hai Hu

**Affiliations:** 1 Windber Research Institute, Windber, PA; 2 Walter Reed Army Medical Center, Washington DC

## Abstract

We developed a quality assurance (QA) tool, namely microarray outlier filter (MOF), and have applied it to our microarray datasets for the identification of problematic arrays. Our approach is based on the comparison of the arrays using the correlation coefficient and the number of outlier spots generated on each array to reveal outlier arrays. For a human universal reference (HUR) dataset, which is used as a technical control in our standard hybridization procedure, 3 outlier arrays were identified out of 35 experiments. For a human blood dataset, 12 outlier arrays were identified from 185 experiments. In general, arrays from human blood samples displayed greater variation in their gene expression profiles than arrays from HUR samples. As a result, MOF identified two distinct patterns in the occurrence of outlier arrays. These results demonstrate that this methodology is a valuable QA practice to identify questionable microarray data prior to downstream analysis.

## Introduction

Gene expression profiling can help in identifying potential biomarkers to aid cancer classification in addition to traditional pathology-based methods (Golub et al. 1999). For example, more than 200 genes have been shown to be highly associated with breast cancer and may be used to predict the clinical outcome of breast cancer (Hu et al. 2003; Paik et al. 2004). Recently, efforts have been made to identify serum biomarkers for the development of a blood-based gene expression test for the early detection of cancers (Sharma et al. 2005). Generation of high quality and reproducible data requires not only strict quality control (QC) of each experimental step, but also quality assurance (QA) that must be performed after laboratory experiments are complete. QA of raw data is essential because 1) it prevents low quality data from entering into later research analysis, and 2) it provides quick feedback for troubleshooting causes of the failed experiments.

Microarray experiments are complicated processes involving multiple steps. From manufacturing of the arrays to data acquisition by scanning of hybridized arrays, numerous factors at any of these steps can lead to unwanted random or systematic variation in the data. The factors impacting microarray raw data can be technical, instrumental and computational (Shi et al. 2004). QA of microarray data is usually conducted at two levels: spot level and array level. To assess the quality at the individual spot level several methodologies have been proposed (Hautaniemi et al. 2003; Chen et al. 2002; Wang et al. 2001; Tran et al. 2002; Sauer et al. 2005). These algorithms usually assign a quality score to a spot based on properties or descriptors related to that spot. Unreliable data points are simply excluded from subsequent analysis. However, not all bad data points can be detected in this way. For example, the levels of decaying transcripts in the sample may be measured correctly but still reflect artifacts. In such cases, it is necessary to assess variation between arrays. To date, however, few publications are available on QA of microarray data at the array level. Traditionally, visual inspection of array images and scatter plots have been used to reveal problematic arrays. Burgoon et al. described an approach to compare arrays based on gross statistical features of datasets (Burgoon et al. 2005). Model et al. used PCA and multivariate statistics process control to detect outlier arrays (Model et al. 2002).

It is important to identify outlier arrays for microarray data analyses. The majority of statistical strategies for differential analysis of gene expression are derived from t-test or ANOVA based analyses (Amaratunga et al. 2004). A limitation to such approaches is that they depend upon accurate estimation of sample variance. However, the small sample sizes used in typical microarray experiments result in unreliable estimation of variance. The situation worsens in the presence of outlier arrays. Even with the introduction of statistical strategies such as Significance Analysis of Microarray (SAM) (Tusher et al. 2001) which was designed to correct unstable variance estimates, this problem of unreliable variance estimation is still only partially alleviated. In this paper, we tackle the problem by adopting objective QA criteria at the whole array level so that outlier arrays can be flagged and treated differently in the downstream analysis. This approach will likely enhance the reliability of the differential gene expression analysis. In essence, our approach is an examination of the consistency among the arrays.

## Experimental Procedure and Data Acquisition

### Blood Samples and RNA Extraction

The PAXgene blood RNA extraction system (PreAnalytiX, Hombrechtikon, Switzerland) was used to collect blood and extract RNA. The system consolidates and integrates the key steps of whole blood collection, nucleic acid stabilization, and RNA purification. It reduces the unpredictability associated with RNA processing and provides enhanced accuracy of intracellular RNA analysis.

Blood samples were collected from donors enrolled in the Clinical Breast Care Project following IRB-approved protocols. 2.5 mls of blood were drawn by qualified individuals directly into PAXgene tubes. The PAXgene tubes contain an additive that stabilizes cellular RNA and prepares the samples for RNA purification. The samples were left in the PAXgene tubes overnight at room temperature and then stored at −20°C or −80°C until use.

Next, tubes were removed from freezer and allowed to warm to room temperature overnight. Total RNA was extracted using the PAXgene blood RNA kit in accordance with the supplier’s instructions. The procedure started with a centrifugation step to pellet nucleic acids in the PAXgene tubes. The pellet was washed, resuspend, and incubated in optimized buffers containing Proteinase K to digest proteins. Residual cell debris was removed by centrifugation, and the resulting supernatant was transferred to a fresh microcentrifuge tube. Ethanol was added and mixed by vortexing. The lysate was then applied to a PAXgene RNA spin column and a brief centrifugation was used to remove contaminants while RNA was selectively bound to the silica-gel membrane of the column. Remaining contaminants were removed by three wash steps, and pure RNA was eluted using nuclease-free water. To denature the RNA, which was essential for maximum efficiency in downstream applications including cDNA synthesis, the eluate was incubated briefly at 65°C in a heating block. Following incubation, RNA samples were chilled immediately on ice. RNA concentration and purity was determined using a NanoDrop (Agilent, Foster City, CA) which provides absorbance measurements at 260nm and 280nm. The A_260_/A_280_ ratio must be above 1.7 for use in microarray experiments. RNA quality was further checked using Agilent Bioanalyzer Nano Chip (Agilent, Foster City, CA). All extracted RNA must have a 28S/16S ratio greater than 1.5 or a RNA Integrity Number (RIN) above 5 to pass this QA measure.

### CodeLink Microarray and Hybridization

We used CodeLink Bioarrays (GE HealthCare, Piscataway, NJ) for microarray experiments. CodeLink utilizes a one-color labeling and detection method based on biotin-labeled cRNA. Human 20K bioarray targets 19881 well-annotated human genes. Each transcript is represented by a 30-mer probe which is designed to conserve exons across the transcripts of targeted genes. Total RNA from each donor was linearly amplified and hybridized to the CodeLink 20K human bioarray following the manufacturer’s recommended protocol. In brief, 1 μg of total RNA together with control bacterial mRNA was used to synthesize the first strand cDNA by reverse-transcription, followed by the second strand cDNA synthesis. After purification of these double-strand cDNA, *in vitro* transcription was carried out to generate cRNA followed by biotin-labeling. An aliquot of these labeled cRNA was run on Agilent’s bioanalyzer for qualification and quantization. A total of 10 μg of high quality cRNA was fragmented and then hybridized to CodeLink Bioarrays. If a sample did not yield enough cRNA or cRNA failed the quality control, the experiment would restart from cRNA amplification from total RNA. After hybridization, arrays were washed, stained with Cy5 dye then scanned using ScanArray 5000 (Perkins Elmer, Wellesley, MA).

### Data Acquisition and Process

Scanned images were analyzed using CodeLink Expression software to generate raw data. We manually checked all the grids to be sure that no miss-griding occurred. Results were stored in a data warehouse internally developed for the integration of clinical, genomic and proteomic data. Perl was used to retrieve and preprocess data for the statistical analysis performed by R along with BioConductor. Spotfire (Spotfire, Inc., Somerville, MA) was the visualization tool for the current study.

## Experimental Datasets

Two groups of microarray datasets were used in this study: 35 arrays of a human universal reference (HUR) RNA sample (Stratagene, La Jolla, CA) and 185 arrays of human blood samples. An HUR sample was included in each microarray experiment as a control for systematic variation from experiment to experiment. These HUR experiments were carried out from September, 2003 to December, 2004. This dataset was used to validate our QA methods and to gain a general assessment of the reproducibility of our experiments. The human blood samples were composed of whole blood from 71 patients with benign breast lesions, whole blood from 82 patients with invasive breast cancer and whole blood from 30 normal controls with no known breast disease. The human blood samples were subjected to microarray experimentation from July, 2003 to August, 2004.

In this study, only a subset of the 20470 probes on a standard 20K array was used for QA analysis. Probes with median raw intensity across the array group (e.g. HUR or human blood samples) below 200 or above 10000 were excluded. Based on our experience, we believe that intensity below the 200 was too close to the background level; thus, the signal measurement might not be reliable. On the other hand, intensity above 10000 fell in the saturation range of the scanner and thus did not effectively reflect the difference in the signal strength. Of the 7080 probes which satisfied these criteria, only good data points, as judged by CodeLink spot quality measures, were included in our QA analysis. The selected datasets were then normalized by quantile normalization across the array group. All subsequent QA analyses were performed on the data preprocessed in this way.

## Rationale and Algorithm

The CodeLink platform has rules to evaluate the quality of individual spot and to flag problematic data points. CodeLink also provides a QA measure, normalized threshold, to gauge the overall intensity of fluorescent signal level on an array. A “too low” or “too high” signal level usually indicates systematic errors in the experiment, and results in rejection of the whole array.

However, these QA measures only focus on a single array without considering its relationship with other arrays. We believe that it can be informative to view individual arrays as a whole against a group of arrays. By monitoring gross changes in a dataset of whole arrays, we expect to identify outlier arrays which are significantly different from other arrays. Our approach is to compare statistics of an array to those of other arrays and look for arrays which are dissimilar to the majority of arrays in the whole group. These “unusual” arrays are candidates for failed arrays which will be investigated further. It should be noted that this rationale is based on two assumptions. First, all samples for comparison are similar enough so that the expression levels of most genes are similar among the samples. When these samples are subjected to the same experimental protocol, it is expected that the gross expression profiles are the same for all the samples. Second, we are confident that most of our array data are of high quality thus consistent with each other. Therefore, a few “unusual” arrays stand out as suspected failed arrays.

The HUR sample was obtained commercially. The manufacturer strived to reduce differences between samples from batch to batch. The 185 human blood samples described in this paper were from different subjects. Although there were biological variations among these samples, based on our experience we expected that the variations were not large enough to substantially change the statistical features of the datasets. In addition, all samples were analyzed using the same experimental protocol. Therefore, our samples were suited to the QA approach described above.

Two statistical indices were used for array comparison. The Pearson correlation coefficient was computed for each and every pair of arrays in the analysis. A collection of the correlation coefficient values was arranged in a table (Table 2) to show similarity and consistency between arrays. An outlier array was expected to have low correlation with other arrays. The other statistical index was the percentage of outlier data points on an array. Here an outlier data point was in the context of all data points of a specific probe across the array group. We used resistant z-score (Amaratunga et al. 2004, pp. 78) to identify an outlier. The resistant z-score is defined as:

$$z_{i}=\frac{X_{i}-\tilde{X}}{\tilde{s}}$$

where *X̃* and *s̃* are the median and MAD (median absolute deviation). As its name implies, a resistant z-score is resistant to outliers’ influences since the median and the MAD are used for calculation. It can tolerate up to 50% outliers without being distorted. A resistant z-score was computed for each data point. A data point with the absolute value of its resistant z-score larger than a preset threshold was designated as an outlier. In this study, we chose to set the threshold to 3 based on our experience with our datasets. We found that this threshold could distinguish outlier arrays from non-outlier arrays. We observed that the more an array differed from other arrays, the more outlier data points were detected on that array. Therefore, the percentage of outlier data points could be used as an indicator for an outlier array. This algorithm using correlation coefficient and percentage of outlier spots was implemented as an R function: microarray outlier filter (MOF), which ranks the arrays by their possibility of failure based on these two statistical indices.

## Visualization and Analysis in Spotfire

Correlation coefficients were displayed in a correlation table. All visualizations, including the heat map of the correlation table, the heat map showing the percentage of outliers, scatter plots between arrays, and images of spatial distribution of spots on arrays, were developed in Spotfire. Clustering of the correlation table was done by complete linkage hierarchical clustering using Euclidean distance as the similarity measure in Spotfire. To compare an array with a group of similar arrays in a scatter plot, a model array was constructed by taking the median of all intensity values of the same probe across all the arrays in that array group, as the value for that probe on the model array. Thus, the model array could be used to represent the group of arrays. To visualize the locations of probes on an array, a 2-D plot was constructed using local coordinates of the probes on the array as provided by CodeLink.

## Results

### Identification of HUR Outlier Arrays

We applied our QA measures to the 35 HUR sample arrays which were expected to be highly consistent. The purpose of analyzing HUR data was to test and validate our QA measures. A correlation coefficient was computed for each pair of the 35 arrays. The average correlation coefficient for an array was used to assess the similarity of the array to other arrays. The percentage of outlier spots on each array was also calculated to serve as an additional indicator of “abnormal” arrays. Arrays with low correlation with the majority of other arrays and/or a higher percentage of outliers were suspected as outlier arrays. There were three arrays (T00225133, T00237520 and T00245878) which had substantially lower correlation to other arrays. These three arrays also generated more outlier data points than other arrays (Table 1). The average correlation coefficient among them was only 0.32. This fact was contradictory to the expectation that HUR arrays should perform consistently. Thus, these three arrays were flagged as failed arrays. Later, we found that two of these arrays, T00225133 and T00245878, had been independently determined as failed arrays by the laboratory based on the median raw intensity—in fact these two arrays were arrays for testing purpose in the laboratory and we intentionally started this QA project with all the available arrays. T00225133 and T00245878 gave median raw intensities of 800 and 25.5, respectively, while normal median raw intensity was expected to be around 100. According to the experimental protocol, these two arrays should be discarded. It was very encouraging that the results by our QA approach were confirmed by independent laboratory measures. At the end our approach flagged one previously unsuspected array as a failure.

### Clustering of HUR Arrays

**Figure 1:**
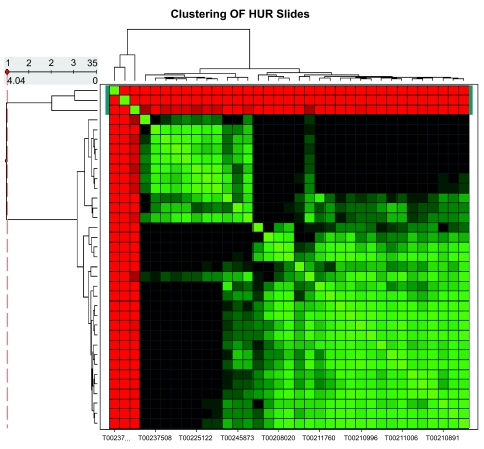
Clustering of the HUR arrays. The correlation coefficient table for the 35 HUR arrays was clustered by hierarchical clustering and displayed as a heat map using Spotfire. From red color to green color, correlation coefficient increases. The 3 outlier arrays were clustered in red.

Correlation coefficients of all array pairs were arranged into a correlation table (Table 2) which was visualized as a heat map in Spotfire. When the correlation table was rearranged by clustering the arrays, we observed that, excluding the three obviously bad arrays described above, the good arrays could be grouped into two main clusters (Figure 1). One cluster contained 11 arrays and the other contained 21 arrays. Arrays in the smaller cluster had lower average correlation and higher percentage of outlier spots than arrays in the larger cluster. In general, arrays with overall low correlation coefficient also contained more outlier points, indicating that correlation coefficient and percentage of outliers produced consistent results. This fact increased our confidence in using the two statistical indices in our QA analysis.

We observed that arrays within a cluster typically had high correlation with each other while arrays between the two clusters displayed poor correlation (Fig. 1). In details, the average correlation for arrays in the smaller cluster was 0.92, and that for the larger cluster was 0.93, whereas the average correlation of arrays between the two clusters was 0.83. These observations demonstrated that while the clustering of the arrays into two groups was not an artifact, the difference between the two clusters was not large at all which was what one would expect when the experiments were performed with the same sample. Further study of the data using scatter plots showed that arrays within a cluster displayed tighter distributions than scatter plots of arrays between the clusters, and the latter contained some spots scattering from one side of the diagonal direction (data not shown). Since the difference between the two clusters was not large, and the sizes of the two clusters were comparable, we did not think it was appropriate to eliminate one group as outlier arrays.

### Spatial Bias of Data Points on HUR Outlier Arrays

**Figure 2:**
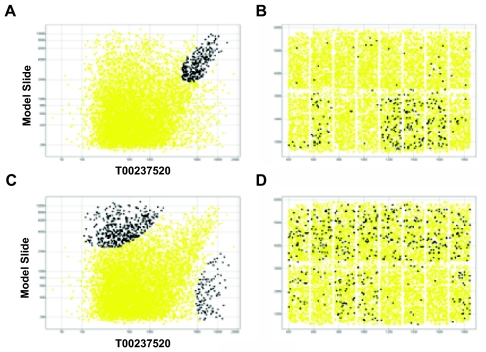
Biased spatial distribution of spots on array T00237520. (A) Scatter plot of log transformed intensity values with the x-axis for the values from this array and the y-axis for those from the model array. The highlighted spots (in black) were probes showing consistent and strong signals (intensity > 2000) in both arrays. (B) The physical locations of the probes on the array. Probes highlighted in (A) were more focused in 4 blocks (black dots). (C) Scatter plot similar to (A) but now the highlighted probes were those whose signal is strong on one array but weaker in the other. (D) Those probes of inconsistent performance were distributed mostly on the other 12 blocks.

Filtering out low-quality arrays helps prevent misleading results in the subsequent data analysis. At the same time, QA analysis can benefit the laboratory by providing clues to possible causes to failed arrays. We set off to inspect the three outlier arrays more closely hoping to gain deeper insight into what might have gone wrong with these arrays by using scatter plots of signal intensity and a spatial distribution plot of the probes on the array (Fig. 2). In Figure 2A, the data points in the scatter plot of array T00237520 and the model array representing the 32 good arrays spread out loosely, demonstrating discrepancy between many data points on array T00237520 and the corresponding data points on the model array. When we displayed the locations of probes whose values on the model array were above 2000, an interesting spatial pattern appeared. Probes whose intensity values were comparable on both array T00237520 and the model array were concentrated in 4 blocks on the array (Fig. 2B). Probes with different intensity values on both arrays were mainly located in the remaining 12 blocks (Fig. 2C and D). Similarly, data points on array T00245878 matching corresponding data points above 2000 on the model array were also found mostly in 6 blocks while inconsistent data points were mostly located in the other 6 blocks on array T00245878 (data not shown). No spatial bias of spot distribution was found for probes with intensity lower than 2000 on the model array (data not shown). Therefore, at least part of the questionable data on arrays T00237520 and T00245878 could be attributed to problems in specific blocks within the array. Intriguingly, problematic blocks were not always the same blocks on these two failed arrays, suggesting the problem occurred more randomly than systematically. The scatter plot of array T00225133 and the model array took a different form than those between the model array and array T00237520 or array T00245878. We believe that there were other causes to the failure of array T00225133.

### Identification of Human Blood Outlier Arrays

**Figure 3:**
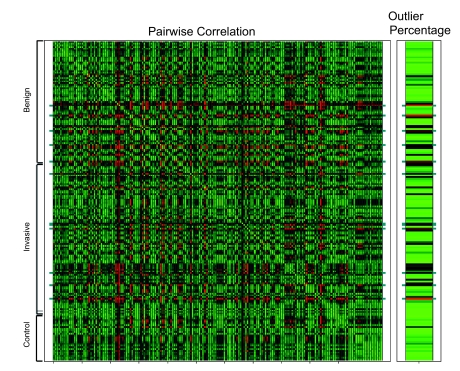
Visualization of correlation between the 185 human blood samples (left panel) and percentage of outlier spots on each of these arrays (right panel). The correlation table of the human blood samples was displayed as a heat map with the red color representing low correlation and green color showing high correlation between a pair of arrays. The percentages of outlier spots for each array were also shown in a heat map on the right with the red and green color standing for high and low percentage of outlier spots, respectively. The arrays were in the same order from top to bottom in both heat maps and the subject categories were shown on the left side of the figure. Marked in dark green were 12 arrays that were flagged as failed ones as described in the text.

The dataset of 185 human blood samples was analyzed the same way as the HUR sample arrays. It could be seen that some arrays had overall poor correlation with other arrays (Figure 3). The average correlation coefficient for one array with the rest 184 was in the range of 0.69 to 0.91. Percentage of outlier points on the arrays spanned from 0.3% to 23.8%. The arrays were then sorted from most likely to least likely to be an outlier array according to either the average correlation coefficient or the percentage of outlier points, respectively. Twelve arrays appeared in the top 30 in both lists, and were designated as suspected failed arrays. Among them, four samples were from patients with benign breast lesions while eight samples were from patients with invasive breast cancer. So the outlier arrays were not a reflection of the disease severity. We also investigated the association between the outlier arrays and the following factors: lots of arrays, lots of PAXgene tubes, dates of the experiments performed and individuals who performed the experiments. No apparent association was found.

## Discussion

We performed QA analysis on two groups of microarray datasets. The HUR data was more homogeneous. It was used to test our QA approach employing correlation coefficient and percentage of outlier spots as the QA measures. Having validated the QA approach, we performed the QA analysis in the same way on the dataset of 185 human blood samples and flagged 12 arrays as candidates for failed arrays. We recommended that microarray experiments with these arrays be repeated. Furthermore, using images of spot distribution reconstructed in Spotfire, we confined problematic areas in certain blocks on two failed arrays.

Although we demonstrated that our QA measures were effective in identifying low-quality arrays, it should be noted that it is important that the samples for the QA analysis are of similar expression profile in general, and the experiments are conducted using the same protocol. Following these rules ensures that outlier arrays detected by our approach were not due to normal variation in expression profile. For example, we checked correlation between expression arrays done on samples from different mouse tissues and found that correlation in array expression between the tissues was so low that clustering of the correlation table was strictly dictated by tissue type (data not shown). In such a scenario, a few highly similar outlier arrays were, instead of failed experiments, just normal expression profiles which differ from profiles of other samples. Such distinct categorization was not seen among the human blood samples, indicating variation among our samples was not large enough to invalidate this QA approach. We will not be surprised, however, that a solid human tissue may display a different expression profile and thus is flagged by our QA measures as different from human blood samples.

Essentially, our QA measures check consistency between experiments. It is not a natural deduction that outlier arrays are always failed arrays. It is possible that the data from outlier arrays are good but other data are consistently wrong. One should always be cautious in rejecting outlier arrays as failed arrays. In our experiments involving the HUR sample, the outlier arrays were not consistent among themselves, making it impossible that all the outlier arrays were good. Therefore, we think that the outlier arrays were truly failed arrays. Based on our experience with the HUR sample, we have confidence in the data quality of most of our human blood samples. Outlier arrays are likely caused by failed experiments although other causes can not be ruled out, e.g. the samples are biologically heterogeneous. It is still an open question whether the outlier arrays may represent some unusual samples, especially in the case of our human blood samples, the difference between the outlier arrays and the other arrays were not as dramatic as in the HUR arrays. More replicates of the outlier arrays should be done to address this issue.

Another issue in the application of our QA approach is how to set the threshold to reject an array. We do not think there is a universal standard. Even with the same data, correlation coefficients can change with the number of probes involved in the QA analysis. Importantly, the thresholds for the average correlation coefficient and the percentage of outlier spots should be adjusted when arrays of unknown quality are analyzed. Thresholds should be established empirically. Familiarity with historical data plays an important role in threshold decision-making. Our QA method is designed to provide the likelihood of possible problems on an array. We inspect potential problems starting from the top of the lists of the potentially problematic arrays reported by MOF. Down the list we usually see the severity of problems decreasing. The setting of the threshold depends on the nature of samples, the goal of analysis and even the availability of resources, etc. Of the 35 HUR RNA experiments, 3 arrays significantly differed from the other arrays and were also different among themselves in regard to the average correlation coefficient and percentage of outlier spots. In this case, it was logical to filter out only these three arrays as outlier arrays. However, with the 185 human blood samples, we did not see such a clear cut dividing of the arrays into outlier and normal arrays. Twelve arrays were flagged as problematic arrays not only because these arrays had relatively low correlation with other arrays and a high percentage of outlier spots but also because other techniques such as scatter plots and clustering provided additional evidence. It should be stressed that our QA approach just evaluates the likelihood of failure for each array. Not necessarily every batch of arrays must contain failed experiments.

Two statistics are employed in our QA approach. With the HUR arrays, both statistics generated highly consistent results. With the human blood samples, there was overlap and discrepancy between outlier arrays screened separately by correlation coefficient and percentage of outlier spots. Not every array which had poor correlation with the other arrays contained more outlier spots. This was not surprising because human blood samples were more heterogeneous than the HUR samples, and there were more human blood sample arrays than the HUR sample arrays in the current study. Intrinsically, the two statistics do not measure exactly the same properties of a dataset. It is natural they may give different results when the situation becomes complicated. Actually, it is an advantage that these two statistics can confirm and compliment each other. Abnormal arrays detected by either one of the QA measures warrant further investigation.

For the correlation coefficient, a minimum of three arrays are needed to identify an outlier array mathematically. However, larger numbers of arrays can give more reliable results. Historical data can be used if insufficient arrays are available. Even though the data may come from different projects, they can be pooled for this QA analysis as long as the two assumptions laid out in Rationale and Algorithm Section are satisfied.

Microarray technology has become more and more mature in the last several years. Technical replicates are rarely used, and biological replicates are counted on to reach a biological conclusion (Allison et al. 2006). However, microarray experiments may still fail due to a variety of reasons including operating errors. Our approach evaluates microarray data quality based on the statistics of the whole array and flags arrays of low quality. It is not always necessary to discard the flagged arrays since at times a substantial amount of good data points can still be salvaged by conducting analysis at the probe level (Hautaniemi et al. 2003; Sauer et al. 2005). It is also possible to make use of the data from arrays of low quality by down-weighting them in the whole data analysis (Ritchie et al. 2006). We expect that by combining our approach with other complementary approaches, a user can not only identify arrays of low quality, but also make the best use of the data generated from all the microarrays.

## Figures and Tables

**Table 1:** Average correlation coefficient and percentage of outlier points for the 35 HUR arrays.

**Array**	**Av Cor**	**Otlr %**	**Array**	**Av Cor**	**Otlr %**	**Array**	**Av Cor**	**Otlr %**
T00245878	0.3	42.23	T00209832	0.8	1.85	T00211006	0.84	0.37
T00237520	0.22	40.56	T00205609	0.78	1.71	T00216482	0.82	0.37
T00225133	0.49	28.21	T00211750	0.81	1.29	T00208091	0.84	0.27
T00208035	0.76	11.11	T00211760	0.81	1.16	T00208342	0.83	0.26
T00237506	0.78	5.32	T00210855	0.83	1.14	T00210907	0.84	0.23
T00208021	0.76	4.27	T00208020	0.8	1.03	T00208057	0.82	0.22
T00237505	0.78	4.16	T00216483	0.83	0.95	T00210996	0.83	0.19
T00237508	0.78	4.07	T00207911	0.83	0.91	T00210869	0.84	0.18
T00236213	0.79	3.7	T00209843	0.82	0.84	T00210856	0.83	0.16
T00245873	0.82	2.75	T00210891	0.83	0.72	T00207898	0.82	0.11
T00225122	0.79	2.63	T00208076	0.81	0.69	T00210880	0.83	0
T00209817	0.8	2.34	T00216489	0.83	0.68			

The average correlation coefficient for each array is computed by averaging the correlation coefficients of that array with every other array. Percentage of outlier spots on an array was computed by dividing the number of outlier spots by the total number of probes involved in the analysis. Data points with resistant z-score below −3 or above 3 were counted as outlier spots.

**Table 2:** Correlation coefficient table for selected HUR arrays.

**Array**	**B_0.00223_**	**B_0.00553_**	**B_0.00215_**	**B_1.00083_**	**B_1.00110_**	**B_0.00170_**
B_0.00223_	1.00	0.85	0.87	0.78	0.90	0.87
B_0.00553_	0.85	1.00	0.86	0.93	0.83	0.83
B_0.00215_	0.87	0.86	1.00	0.87	0.92	0.90
B_1.00083_	0.78	0.93	0.87	1.00	0.81	0.83
B_1.00110_	0.90	0.83	0.92	0.81	1.00	0.88
B_0.00170_	0.87	0.83	0.90	0.83	0.88	1.00
